# Soluplus^®^ polymeric nanomicelles improve solubility of BCS-class II drugs

**DOI:** 10.1007/s13346-022-01182-x

**Published:** 2022-05-23

**Authors:** Rosario Pignatello, Roberta Corsaro, Angela Bonaccorso, Elide Zingale, Claudia Carbone, Teresa Musumeci

**Affiliations:** 1 Department of Drug and Health Sciences, University of Catania, 95125 Catania, Italy; 2 NANOMED - Research Centre on Nanomedicine and Pharmaceutical Nanotechnology - University of Catania, 95125 Catania, Italy

**Keywords:** Micelles, Aqueous solubility, LogS, Nanomedicine, Drug delivery

## Abstract

The issue of poor aqueous solubility is often a great hitch in the development of liquid dosage forms for those drugs that the Biopharmaceutics Classification System (BCS) includes in classes II and IV. Among the possible technological solutions, inclusion of the drug molecule within polymeric micelles, and particularly nanomicelles, has been proposed in the last years as a valid strategy. Our attention has been recently attracted by Soluplus^®^, an amphiphilic polyvinyl caprolactam-polyvinyl acetate-polyethylene glycol graft copolymer able to form small and stable nanomicelles. The aim of this study was to characterize Soluplus^®^ nanomicelles to enhance the apparent solubility of three model APIs, categorized in BCS class II: ibuprofen (IBU), idebenone (IDE), and miconazole (MIC). Drug-loaded Soluplus^®^ micelles with a mean size around 60–70 nm were prepared by two methods (direct dissolution or film hydration method). The prepared nanosystems were characterized in terms of mean particle size and Zeta potential, physical stability, drug solubility, and in vitro drug release. The solubility of the tested APIs was shown to increase linearly with the concentration of graft copolymer. Soluplus^®^ can be easily submitted to membrane filtration (0.2 µm PES or PTFE membranes), showing the potential to be sterilized by this method. Freeze-drying enabled to obtain powder materials that, upon reconstitution with water, maintained the initial micelle size. Finally, viscosity studies indicated that these nanomicelles have potential applications where a bioadhesive material is advantageous, such as in topical ocular administration.

**Graphical abstract:**

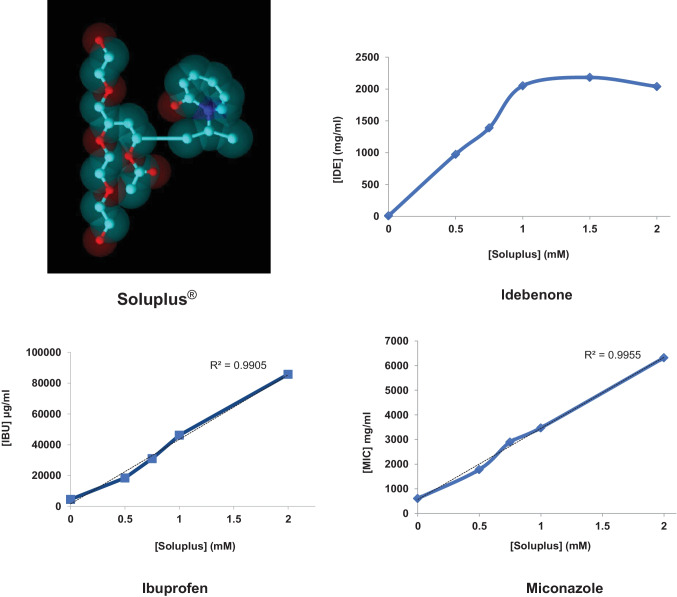

**Supplementary information:**

The online version contains supplementary material available at 10.1007/s13346-022-01182-x.

## Introduction

Drug solubilization has drawn attention in recent years because large numbers of active pharmaceutical ingredients (API) often fail in formulation development due to their limited solubility and bioavailability [1].

The Biopharmaceutics Classification System (BCS) categorizes drug molecules into four groups based on their solubility and permeability profiles:

- Class I: high permeability, high solubility compounds
- Class II: high permeability, low solubility compounds
- Class III: low permeability, high solubility compounds
- Class IV: low permeability, low solubility compounds

Up to 50% of all the authorized drug are categorized in classes II and IV [2]. For molecules belonging to classes II and IV, the main goal in formulation development is increasing their solubility [3].

Solubility question concerns various therapeutic means, such as eye drops, buccal and intranasal solutions, injections, and, in general, those systems in which stable aqueous formulations are required. Drug modifications such as salts, co-crystals, and polymorphs are sometimes used to increase the solubility of these molecules [4]. Other proposed strategies include complexation and formation of solid dispersions [5, 6].

More recently, various colloidal drug delivery systems have been proposed to overcome the limits towards in vivo applications of such compounds. Among them, a valid approach to improve the solubility of APIs is formulation of micelles, using macromolecules that self-assemble into ordered structures which able to host hydrophobic drug molecules in the interior domain, and thereby a higher apparent solubility in aqueous media is attained [7–10].

Polymeric micelles have drawn a large attention thanks to their technological features: preparation methods are simple, including an easy industrial scalability, they are highly biocompatible, and can efficiently encapsulate poorly soluble and lipophilic compounds, delivering them in the body also with a targeting potential [8–10].

Micelles are colloidal dispersions belonging to the large family of dispersed colloidal systems, composed of a dispersed phase, distributed within a dispersing medium (continuous phase). In solution, surfactants aggregate to micelles in concentrations above their critical micelle concentration (CMC) forming a colloidal solution. When micelles are diluted below this concentration, they may collapse. CMC is therefore the minimum concentration required by an amphiphilic molecule to begin micellization, and its value is specific for any monomer [11]. Amphiphilic polymers have low CMC values, in the range of 10^−6^ to 10^−7^ mol/l, and this is advantageous for micelles formation and stability, for example, after dilution in the bloodstream. Some materials also show a critical micellar temperature (CMT, also known as Krafft point), e.g., the temperature above or below which the aggregation as micelles or separation into single monomers can occur [12].

In aqueous solutions, a lipophilic compound can be incorporated in the core of micelles, considering that the lipophilic portion of the forming polymer is included in the core and the polar portion formed the shell; the localization of an API molecule inside a micelle is actually dependent on its lipophilicity.

The structure of polymeric micelles is dependent on the polymer chemistry. Spherical or cylindrical micelles can be formed from amphiphilic di-block, tri-block, and graft copolymers when they are in dilute solutions in a solvent that preferentially solvates one of the blocks.

**Fig. 1:**
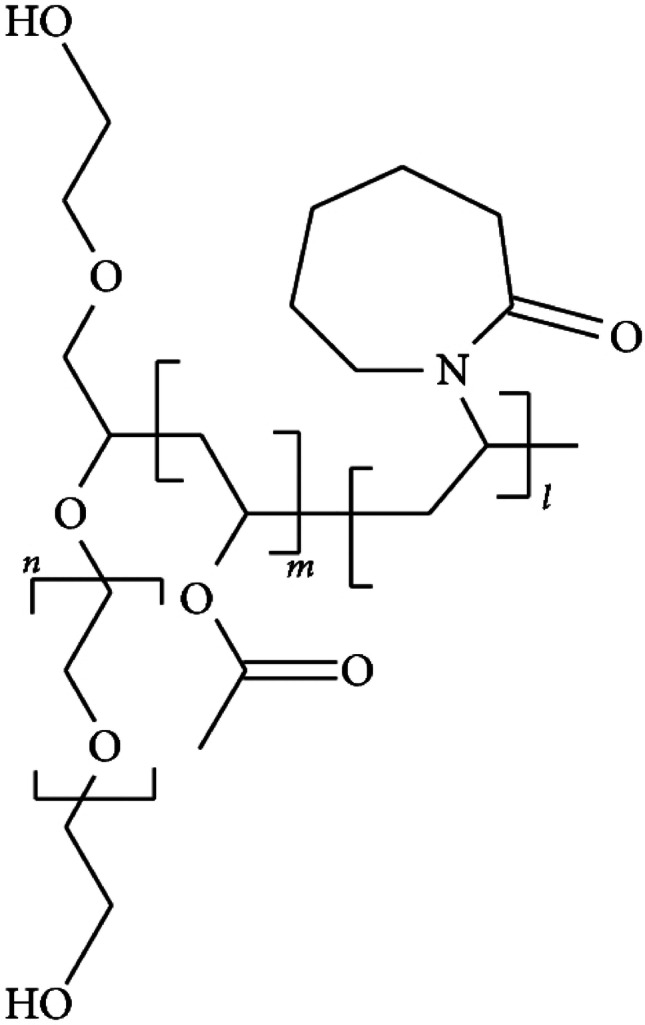
Chemical structure of Soluplus^®^

Among the polymeric materials that in the last years have been investigated for its potentiality in pharmaceutical formulations and drug delivery, Soluplus^®^ has attained great attention. It is a polyvinyl caprolactam-polyvinyl acetate-polyethylene glycol graft copolymer [13] (Fig. 1), having the polyethylene glycol backbone as the hydrophilic part and vinylcaprolactam/vinyl acetate side chains as the lipophilic moiety. Such amphiphilic nature makes it able to form micelles in aqueous solution above the CMC value of 7.6 mg/l [13].

The main physico-chemical properties of Soluplus^®^ are summarized in Tables 1 and 2. Among the various applications, Soluplus^®^ has been proposed as a safe and versatile material to produce nanomicelles in the pharmaceutical field, either alone or in combination with other polymers [14–23].

**Table 1:** Main physico-chemical properties of Soluplus^®^ (source: BASF technical information sheet)

**Chemical composition**	**PEG600/Vinylcaprolactam/vinyl acetate (13/57/30)**
Appearance	White to yellowish free-flowing granules
Molecular weight	90,000–140,000 g/mol (average: 118,000)
Glass transition temperature (Tg)	~ 70 °C
Flow coefficient (Kv value; 1% ethanol)	31–41
Critical micellar concentration (CMC)	7.6 mg/L–7.6 ppm (approx.. 6.5 × 10^−5^ mM)
Minimum ignition energy	10–30 mJ
Lower crystalline solution temperature (LCST)	~ 40 °C

**Table 2:** Solubility of Soluplus^®^ in common solvents [10]

Water	Soluble
Acetone	Up to 50% (w/v)
Methanol	Up to 45% (w/v)
Ethanol	Up to 25% w/v)
Dimethylformamide	Up to 50% (w/v)

The aim of this study was to prepare and characterize Soluplus^®^ nanomicelles as a mean to enhance the apparent solubility of three model APIs, categorized in BCS class II: ibuprofen (IBU), idebenone (IDE), and miconazole (MIC).

**Fig. 2:**
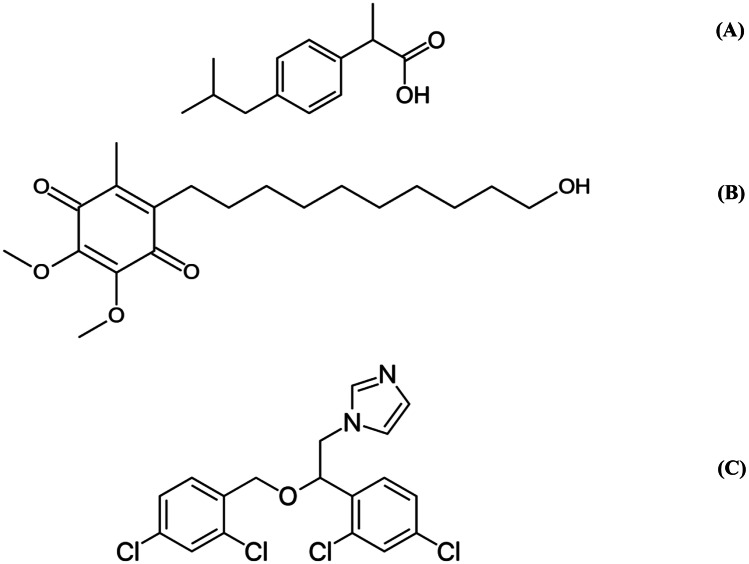
Chemical structure of ibuprofen (**a**), idebenone (**b**), and miconazole (**c**)

IBU (Fig. 2a), a propionic acid derivative, is a prototypical nonsteroidal anti-inflammatory agent (NSAID) with analgesic and antipyretic properties, used for the symptomatic treatment of rheumatoid arthritis, juvenile rheumatoid arthritis, and osteoarthritis. It may be used to treat mild to moderate pain and for the management of dysmenorrhea and to reduce fever. It may be also used i.v. to relieve moderate to severe pain.

IDE [2-(10-hydroxydecyl)-5,6-dimethoxy-3-methyl-1,4-benzoquinone] (Fig. 2b) is a synthetic analogue of coenzyme Q10, a vital cell antioxidant and essential component of the electron transport chain (ETC). It has been proposed that by interacting with ETC, IDE increases ATP production required for mitochondrial function, reduces free radicals, inhibits lipid peroxidation, and consequently protects the lipid membrane and mitochondria from oxidative damage. IDE is currently only authorized by the European Medicines Agency (EMA) for the treatment of visual impairment in adolescent and adult patients with Leber’s hereditary optic neuropathy (LHON), an inherited mitochondrial degeneration of retinal ganglion cells, resulting in acute central vision loss.

MIC (1-[2-(2,4-dichlorophenyl)-2-[(2,4-dichlorophenyl)methoxy]ethyl]-1H-imidazole) (Fig. 2c) is an imidazole antifungal agent used topically and by i.v. infusion. MIC selectively affects the integrity of fungal cell membranes, which have a high ergosterol content and differ in composition from mammalian cells membranes. Table 3 gathers some physico-chemical properties of the three APIs.

**Table 3:** Physico-chemical properties of the used model APIs (source: https://www.drugbank.ca)

**API**	**Property**	**Value**
**Ibuprofen**	Water solubility	0.021 mg/mL
	LogP	3.97
	LogS	−3.99
**Idebenone**	Water solubility	0.00747 mg/mL
	LogP	4.5
	LogS	−4.7
**Miconazole**	Water solubility	0.000763 mg/mL
	LogP	5.86 −5.96
	LogS	−5.7

To assess the influence of the preparation method, drug-loaded Soluplus^®^ nanomicelles were produced by two commonly used approaches, namely direct dissolution and solvent evaporation–thin film hydration method [16]. The produced systems were characterized in term of mean particle size and Zeta potential, physical stability under different storage temperatures, drug solubility, viscosity, and in vitro drug release profile. Membrane filtration of the micelle suspensions was carried out to verify the future possibility of obtaining sterile formulations. A lyophilization study was also performed to prove the possibility of increasing the shelf-life of these systems in the solid state.

## Materials and methods

### Materials

Soluplus^®^ was a kind gift from BASF (Germany). The tested APIs were purchased from the Merck Life Science S.r.l. (Milan, Italy). Solvents were purchased from Exacta + Optech Labcenter SpA (San Prospero, Italy).

### Preparation of blank (unloaded) micelles

Soluplus^®^ solutions were prepared by dissolving the polymer in distilled water, under constant magnetic stirring at room temperature for 48 h. The concentrations studied were 0.5, 0.75, 1, 1.5, 2, and 2.5 mM, respectively, corresponding to a polymer concentration of 5.75, 8.625, 11.5, 17.25, 23.0, and 28.75% (w/v). The upper value in such a range was chosen since Soluplus tends to form highly viscous or gelled solutions at higher concentrations [16], as also shown below in the viscosity studies.

### Preparation of drug-loaded micelles by direct dissolution

The tested drugs were added to 100 mL of a Soluplus^®^ 1.5 mM micellar suspension under constant magnetic stirring for 24 h at room temperature, to reach the following final drug concentrations:

- IDE: 0.1% (w/v) (batch SNM-IDE1)
- IBU: 0.5% (w/v) (batch SNM-IBU1)
- MIC: 0.5% (w/v) (batch SNM-MIC1)

In the case of IDE, a lower concentration was preferred, since greater amounts of this drug resulted in the formation of a turbid suspension.

### Preparation of drug-loaded micelles by solvent evaporation–thin film hydration

In a round-bottomed flask, the required amount of each drug (10 mg IDE, 50 mg IBU, 50 mg MIC) was dissolved in 10 mL of acetone. Soluplus^®^ was added (1.725 g), and the mixture was stirred until a limpid solution was obtained. The solvent was evaporated off by a rotating evaporator at 60 °C for 2–3 h, until a thin film was produced. The vessel was kept under high vacuum overnight and the rehydrated with water (10 mL) under magnetic stirring at 650 rpm at room temperature to achieve a final drug concentration of 0.1% (w/v) (SNM-IDE2) or 0.5% (w/v) (SNM IBU2 and SNM-MIC2) and a 1.5 mM Soluplus^®^ micellar suspension.

### Nanomicelle characterization by photon correlation spectroscopy (PCS)

Mean particle size (Z-Ave), polydispersity index (PdI), and Zeta potential (ZP) were determined by PCS using a Nanosizer ZS90 (Malvern Instruments, UK). Samples were diluted ten-fold with HPLC-grade water before analysis; the reported values are the mean ± S.D. of 90 measurements (3 sets of 10 measurements in triplicate). The ZP values were calculated by the same instrument software from the average values of electrophoretic mobility, using the Smoluchowski equation; values are the mean of up to 3 sets of 100 measurements. The pH values were measured with an XS INSTRUMENTS^®^ model pH 510 pH-meter (OPTO-LAB Instruments S.r.l., Concordia sulla Secchia, Italy).

### Solubility studies

Soluplus^®^ solutions (5 mL) at various concentrations (from 0.5 to 2 mM in water) were placed in centrifugation glass tubes, and an excess of the tested drugs was added. Solubility of the neat drugs in distilled water was also tested. All the dispersions were magnetically stirred for 24 h at 25 °C. After centrifugation at 13,000 rpm and 10 °C for 1 h (SL 16R Centrifuge, Thermo Fisher Scientific, Inc.) to separate the undissolved drug, the absorbance of supernatants, diluted at a 7:3 ratio with either methanol (for MIC and IBU) or ethanol (or IDE), was measured by a GENESYS™ 10S UV–Vis spectrophotometer (Thermo Fisher Scientific, Inc.), using the respective calibration curve previously prepared in methanol for IBU (linear in the range 50–1000 μg/mL, *r*^2^ = 0.9999, *λ*_max_ = 265 nm) and MIC (linear in the range 10–500 μg/mL, *r*^2^ = 0.9989, *λ*_max_ = 230 nm) or in ethanol for IDE (linear in the range 10–500 μg/mL, *r*^2^ = 0.9997, *λ*_max_ = 290 nm). The choice of methanol in the first cases was related to the poor solubility of MIC and IBU in ethanol.

The above data were used to calculate some solubility parameters [25]:

- Molar solubilization capacity (*χ*) (moles of drug that can be solubilized per mol of micellizing copolymer):
  1 $$\chi =\frac{{S}_{tot}-{S}_{w} }{{C}_{copol}-CMC}$$
- Micelle/water partition coefficient (*P*) (the ratio between the drug concentration in the micelles and in the aqueous phase):
  2 $$P=\frac{{S}_{tot}-{S}_{w}}{{S}_{w}}$$
- Molar micelle/water partition coefficient (*MP*) (i.e., the above parameter normalized to 1 M, to remove the dependence of *P* on copolymer concentration):
  3 $$MP= \frac{\upchi\;\cdot \;(1-CMC)}{\mathrm{Sw}}$$
- Gibbs standard-free energy of solubilization that was calculated from the above *MP* values as:
  4 $$\Delta GS=-RT \cdot \mathrm{ln}(MP)$$

In these equations, *S*_tot_ represents the total molar solubility of each API in the micellar solution, *S*_*w*_ is their molar solubility in water, CMC is Soluplus^®^ critical micelle concentration, *C*_copol_ is the copolymer molar concentration in each micelle solution, *R* is the universal constant of gases (*R* = 8.31433 J/mol °K), and *T* was set at 298.15 °K.

### Stability studies

The nanomicellar suspensions were stored in closed glass vials at three different storage conditions (room temperature, 4 °C or 37 °C) and analyzed by PCS after 1, 3, and 6 months. Z-ave and PdI values were recorded and compared with the initial ones.

### In vitro* drug release*

The in vitro release of the three drugs from micelles was investigated by a dialysis bag method, using a Specta/Por^®^ dialysis membrane (MWCO: 3.5 kD) previously soaked overnight in distilled water. One milliliter of each formulation was placed into the dialysis bag and dialyzed against 40 mL of a water–ethanol 70:30 v/v mixture for SNM-IDE1 or water–methanol (70:30 v/v) for SNM-MIC1 and SNM-IBU1. The two different solvents were chosen according to the better solubility of each drug. The systems were kept at 35 ± 1 °C and stirred at 50 rpm min^−1^ using a magnetic stirrer. At predetermined intervals, 2 mL of the external medium were withdrawn and replaced by an equal volume of the same dissolution medium. APIs dissolution curves were obtained analogously, by placing into the dialysis bag 1 mL of an API suspension in water. The taken samples were analyzed by UV spectrophotometry (see above), performing a volume correction for each taken aliquot. Each test was repeated in duplicate.

### Micelle filtration assay

Experiments were carried out to evaluate the future possibility of sterilizing the drug-loaded nanomicelles by 0.2-μm membrane filtration while preserving the same characteristic of fresh nanomicelles. Two different types of sterile syringe filters were tested to choose the most suitable material: Whatman^®^ GD/X 25-mm disposable filters with hydrophilic polyethersulfone membrane (pore size: 0.2 µm) and 13-mm Millex^®^-LG disposable filters with a 0.2 µm hydrophilic Fluoropore™ poly(tetrafluoroethylene) (PTFE) membrane, both purchased from the Merck KGaA, Darmstadt, Germany. The mean size of drug-loaded nanomicelles was measured by PCS immediately after the preparation and following their filtration through the above devices.

### Viscosity studies

The flow behavior of Soluplus^®^ micellar suspensions was determined with a Bohlin CVO programmable rheometer (Malvern Instruments Ltd., Malvern, UK). The test formulation (2 mL) was placed on the cone-plate holder (4° angle, 4 mm diameter), and the angular velocity (shear rate) was set at 5 (1/s).

### Lyophilization

The drug-loaded micellar suspensions were freeze-dried in the absence or in the presence of a cryoprotectant agent, namely trehalose that was tested at two different concentrations, 5% and 15% (w/v). Trehalose was added to 2 mL of each suspension and stirred until completely dissolved. Samples were frozen at −20 °C and freeze-dried for 24 h (Edwards Modulyo, Thermo Fisher Scientific Italia, Rodano, Italy). The resulting powder was then reconstituted with the initial volume of water under gentle hand shaking. The micelle size was then verified by PCS.

### Micelle stability against dilution

IBU-loaded micelle suspensions prepared with 1 and 2 mM Soluplus^®^ (0.5%, w/v of drug) were used for this experiment. Aliquots of each dispersion (60 or 300 µL) were placed into quartz cells containing water to a total volume of 3 mL and kept at 35 °C. The dispersion was shaken at 50 rpm, and the absorbance at 265 nm was registered every 5 min for a total time of 30 min. Each experiment was performed in triplicate. In parallel, IDE-loaded micelles produced with the same copolymer concentrations and containing 0.1%, w/v of drug, were submitted to an analogous dilution assay and analyzed by PCS to measure any change in micelle mean size.

## Results and discussion

### Nanomicelle preparation and characterization

As shown in Table 4, neat Soluplus^®^ forms micelles with a mean size lying in a nanometric range, well falling within the values suitable also for ophthalmic application (< 200 nm) [26]. The size appears to be independent on the polymer concentration, and the samples showed to be highly homogeneous, as proven by the very low PdI values (< 0.1).

**Table 4:** Size analysis (Z-ave), polydispersity index (PdI), and Zeta potential (ZP) of blank Soluplus^®^ nanomicelles aqueous suspensions (means ± S.D.)

**Soluplus**^®^ **concentration**	**Z-Ave (nm)**	**Peak1 (nm)**	**Peak1 area**	**PdI**	**ZP (mV)**
2.5 mM	66.26 ± 0.166	71.37 ± 1.738	100%	0.060 ± 0.027	−0.55 ± 0.03
2 mM	61.22 ± 0.754	64.95 ± 0.936	100%	0.041 ± 0.006	−0.47 ± 0.09
1.5 mM	61.78 ± 1.611	67.11 ± 1.622	100%	0.068 ± 0.022	−0.74 ± 0.31
1 mM	61.03 ± 1.002	64.15 ± 1.617	100%	0.027 ± 0.026	−0.55 ± 0.22
0.75 mM	61.73 ± 1.364	65.34 ± 1.589	100%	0.035 ± 0.006	−0.80 ± 0.07
0.5 mM	63.12 ± 0.764	69.21 ± 1.431	100%	0.091 ± 0.014	−0.87 ± 0.17

A slight negative surface charge was measured, with ZP values ranging from −0.55 to −0.87 mV at all the copolymer concentrations.

Drug-loaded micelles showed mean size values in the same range, regardless of the preparation method, also in this case with a very high size homogeneity (PdI < 0.2) (Table 5). According to these data, blank and loaded micelles can be considered both as nanomicelles (smaller than 100 nm).

**Table 5:** Size analysis (Z-ave), polydispersity index (PdI), and Zeta potential (ZP) of drug-loaded nanomicelles (means ± S.D.)

**Formulation**	**Size analysis**			**PdI**	**ZP (mV)**
	**Z-Ave (nm)**	**Peak1 (nm)**	**Peak1 area**		
**SNM-IDE1**	59.00 ± 1.028	63.06 ± 1.145	100%	0.053 ± 0.011	−2.98 ± 0.683
**SNM-IDE2**	60.60 ± 1.385	65.25 ± 3.108	100%	0.056 ± 0.038	−1.23 ± 0.500
**SNM-IBU1**	50.20 ± 0.145	54.22 ± 1.095	99.5%	0.164 ± 0.022	−2.43 ± 0.235
**SNM-IBU2**	59.05 ± 0.960	64.00 ± 0.673	100%	0.100 ± 0.053	−4.12 ± 0.334
**SNM-MIC1**	55.62 ± 0.039	63.45 ± 3.265	98.8%	0.189 ± 0.008	0.481 ± 0.011
**SNM-MIC2**	56.42 ± 3.537	55.77 ± 4.956	99.9%	0.156 ± 0.030	0.789 ± 0.222

IBU- and IDE-loaded micelles showed a slight negative surface charge, not dissimilar from empty micelles (Table 4); since pure Soluplus^®^ micelles themselves have a slight negative surface charge [25], the absence of change in this parameter upon loading the various APIs would imply their complete allocation within the core of the micelles and not on their surface. In fact, the presence of residual IBU (whose pure aqueous 1% suspension displays a net negative charge, around −60 mV) or IDE (a 0.5% aqueous suspension of it has a ZP of −55 mV) would have increased the ZP value of the micelle dispersions. Conversely, a slight positive ZP value was registered for both the MIC-loaded systems, suggesting that the drug can be partially allocated on the micelle surface.

### Solubility studies

Solubility of IDE in water at 25 °C was very low (0.00747 mg/mL). One aim of this study was to elucidate to what extent APIs concentration can be increased using Soluplus^®^ nanomicelles.

**Fig. 3:**
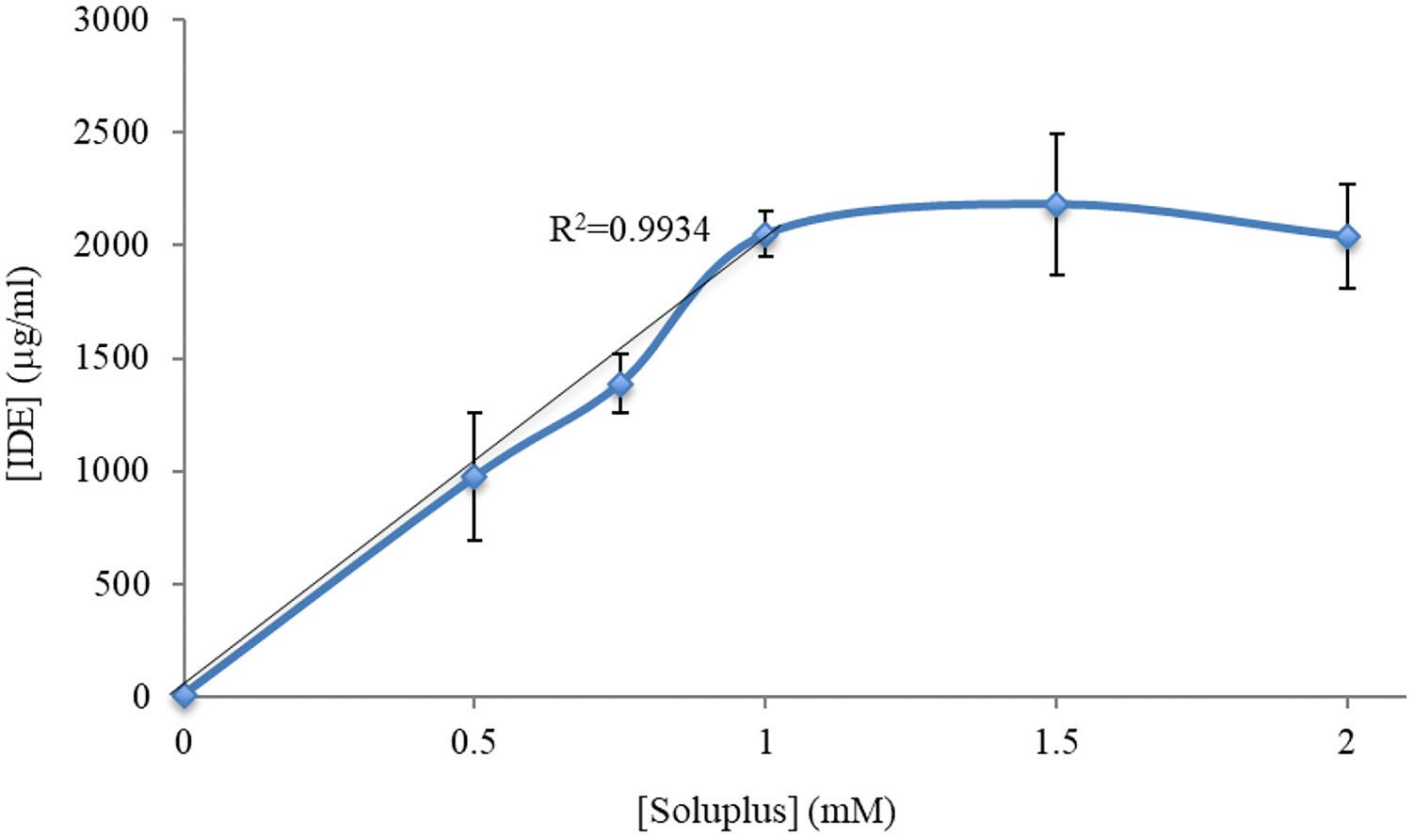
Solubilizing effect of increasing Soluplus^®^ concentrations on IDE (mean ± S.E. of three determinations)

As Fig. 3 shows, the solubilizing effect of Soluplus^®^ micelles of IDE was remarkable. For instance, a 1.5-mM Soluplus^®^ micellar suspension is enhanced by about 300-fold the apparent solubility of the drug in water. A further increase of polymer concentration did not additionally improve the amount of dissolved drug, suggesting a phenomenon of saturation of the micellar structures.

**Fig. 4:**
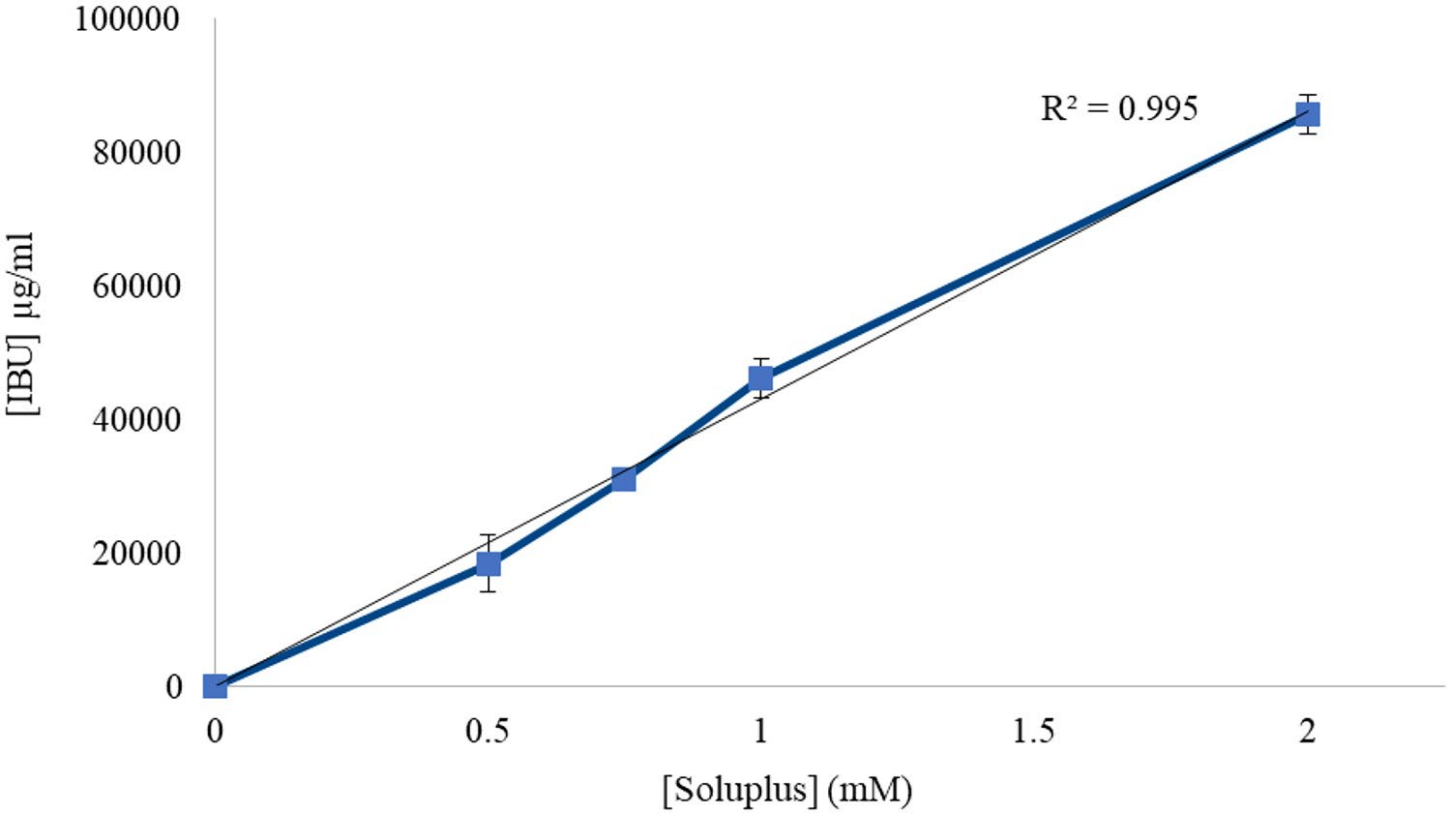
Solubilizing effect of increasing Soluplus^®^ concentrations on IBU (mean ± S.E. of three determinations)

Soluplus^®^ exerted a good solubilizing effect also on IBU: for instance, 2-mM Soluplus^®^ micelles enhanced more than 13-fold the apparent solubility of IBU in water, as shown in Fig. 4.

**Fig. 5:**
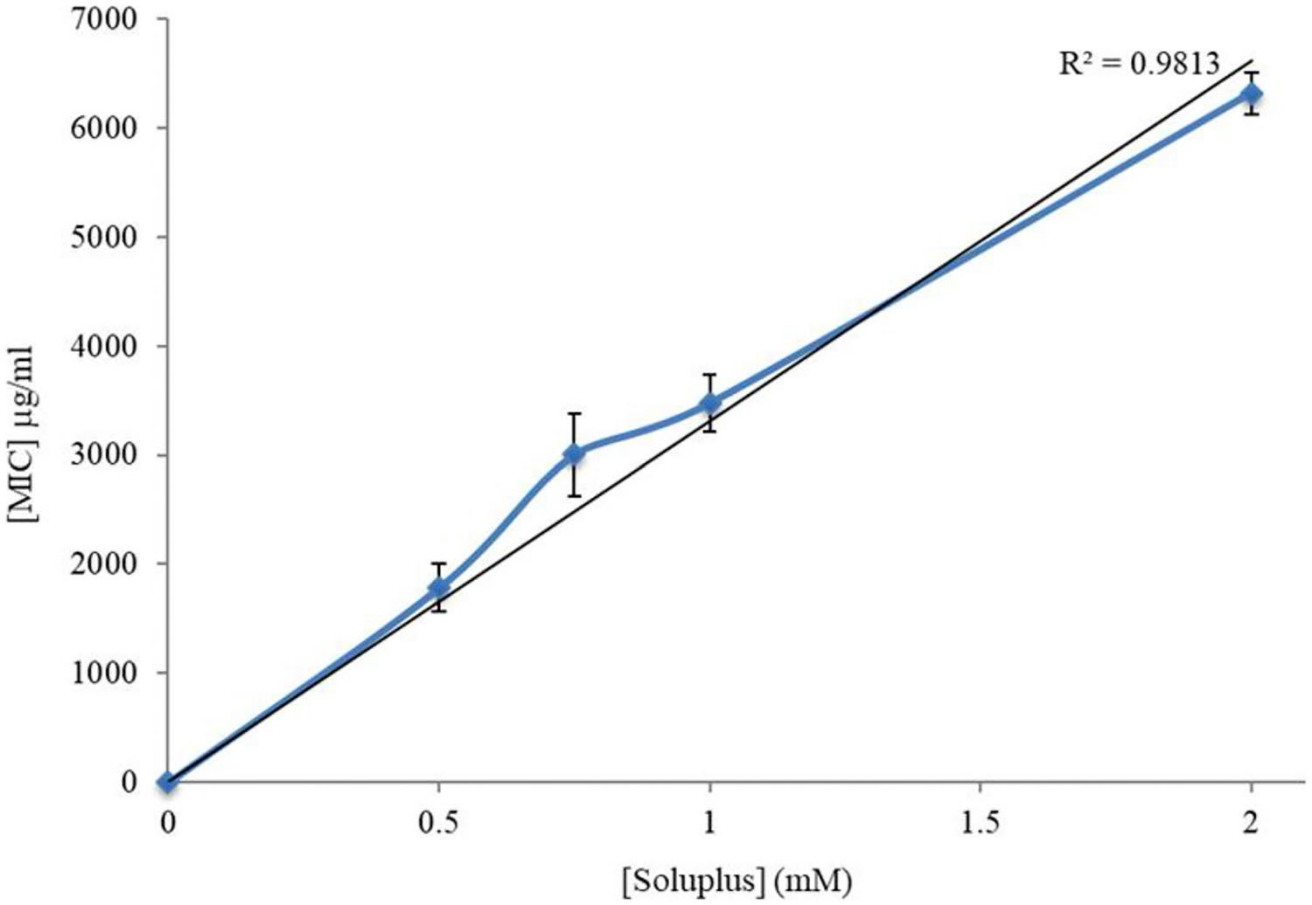
Solubilizing effect of increasing Soluplus^®^ concentrations on MIC (mean ± S.E. of three determinations)

In the case of MIC, the apparent solubility increased linearly within the range of copolymer concentrations tested. For instance, 2-mM Soluplus^®^ micelles enhanced more than tenfold the solubility of MIC in water, as shown in Fig. 5.

The parameters adopted to detail the efficiency of solubilization of the tested APIs in Soluplus^®^ nanomicelles [25] are gathered in Table 6. The behavior of IDE-, IBU-, and MIC-loaded micellar systems was not identical. The molar solubilization capacity *χ* showed a general increasing trend with the increase of Soluplus^®^ concentration, up to a maximum value that corresponds to a copolymer concentration between 0.75 and 1 mM; after that, a net drop of the *χ* value was observed in the case of IDE, indicating that the copolymer is not only involved in forming more micelles but the micelles are formed by more Soluplus^®^ units [25]; for MIC and IBU, conversely, the molar solubilization capacity remained almost constant, suggesting the formation of progressively more micelles containing the host molecules with increasing Soluplus^®^ concentration.

**Table 6:** Solubilization parameters (Eqs. 1–4) of the three tested drugs by Soluplus^®^ nanomicelles, experimentally derived from the dissolution tests. Legend: *χ* molar solubilization capacity, *P* micelle/water partition coefficient, *MP* molar micelle/water partition coefficient, Δ*G*_*S*_ Gibbs standard-free energy of solubilization, *x* molar fraction of drug encapsulated inside the micelles

Soluplus concentration (mM)	IDE solubility (μg/mL)	IDE solubility (mM)	*χ*	*P*	*MP*	Δ*G*_*S*_ (kJ/mol)	*x*
0.5	975	2.878	5.741	384.27	768.60	−14.753	0.852
0.75	1390	4.102	5.460	548.13	730.86	−15.634	0.846
1	2050	6.058	6.051	809.98	809.98	−16.601	0.858
1.5	2185	6.450	4.295	862.45	574.96	−16.757	0.811
2	2040	6.026	3.009	805.69	402.83	−16.588	0.751

Soluplus concentration (mM)	IBU solubility (μg/mL)	IBU solubility (mM)	*χ*	*P*	*MP*	Δ*G*_*S*_ (kJ/mol)	*x*
0.5	18,400	89.195	178.21	890.95	1782.02	−16.838	0.994
0.75	30,980	150.177	200.12	1500.77	2001.07	−18.130	0.995
1	46,166	223.792	223.71	2236.92	2236.92	−19.120	0.996
2	85,750	415.677	207.80	4155.77	2077.82	−20.655	0.995

Soluplus concentration (mM)	MIC solubility (μg/mL)	MIC solubility (mM)	*χ*	*P*	*MP*	Δ*G*_*S*_ (kJ/mol)	*x*
0.5	1785	0.00428	0.0050	1.38	2.756	−0.794	0.0085
0.75	3005	0.00722	0.0072	3.01	4.015	−2.733	0.0095
1	3475	0.00835	0.0065	3.64	3.639	−3.202	0.0083
2	6320	0.01520	0.0067	7.44	3.722	−4.976	0.0075

Analogously, the micelle-water partition coefficient *P* recorded for IDE systems increased up to a 1.5 mM copolymer concentration; afterwards a plateau/tendency to reduction was observed, in line with the dissolution curve measured for this API (Fig. 3), which indicated that, above a certain drug-to-copolymer ratio, the micelles were not able to allocate more IDE molecules. For IBU and MIC systems, conversely, an almost linear positive trend for this parameter was observed, also in this case conform to their dissolution profiles (Figs. 4 and 5). The high positive values of *P*, and of the related molar micelle/water partition coefficient *MP*, clearly indicate that drugs molecules are efficiently incorporated inside the nanomicelle cores. In particular, for all the Soluplus^®^ concentrations tested, IDE was hosted for 85% in the micelles, while more than 99% IBU was present in the polymeric micelles. On the contrary, MIC values of *P* were only slightly above 1, suggesting that a fraction of the added drug remained free (solubilized) in the aqueous medium; the low molar fraction (*x*) values registered for MIC, compared to the other two drugs (Table 6), further confirm the reduced capacity of Soluplus^®^ micelles to solubilize these molecules. The dispersion of a host molecule in a micellar structure is of course a complex phenomenon, resulting from different and concomitant parameters and properties of the host and from the surrounding environment. In particular, the amphiphile-like structure of IDE (Fig. 2b) can raise the hypothesis that these molecules stay not only within the lipophilic micelle core, but someway aligned with the copolymer backbone. Such a behavior, that of course would deserve further analytical confirmation, could explain the upper limit of solubility and micelle/water partition observed for IDE as a function of Soluplus^®^ concentration.

In accordance to Alvarez-Rivera et al. [25], the higher partition coefficients measured for Soluplus^®^ nanomicelles were associated to more negative values of Gibbs free energy of solubilization (Δ*G*_*s*_): this indicates that the solubilization of the APIs in the micelles was a spontaneous process that was thermodynamically supported by the dilution of the drugs within the hydrophobic micelle inner structure.

### Stability studies

The drug-loaded micellar suspensions formulations were kept for 6 months in closed glass vials at different storage conditions (4, 25, and 37 °C) to analyze the possible variations of physico-chemical characteristics over time. The nanomicelles suspensions were found to be stable under the specified conditions; specifically, in terms of mean particle size and PdI, no change was observed in all the tested samples (Tables S1–S3).

**Fig. 6:**
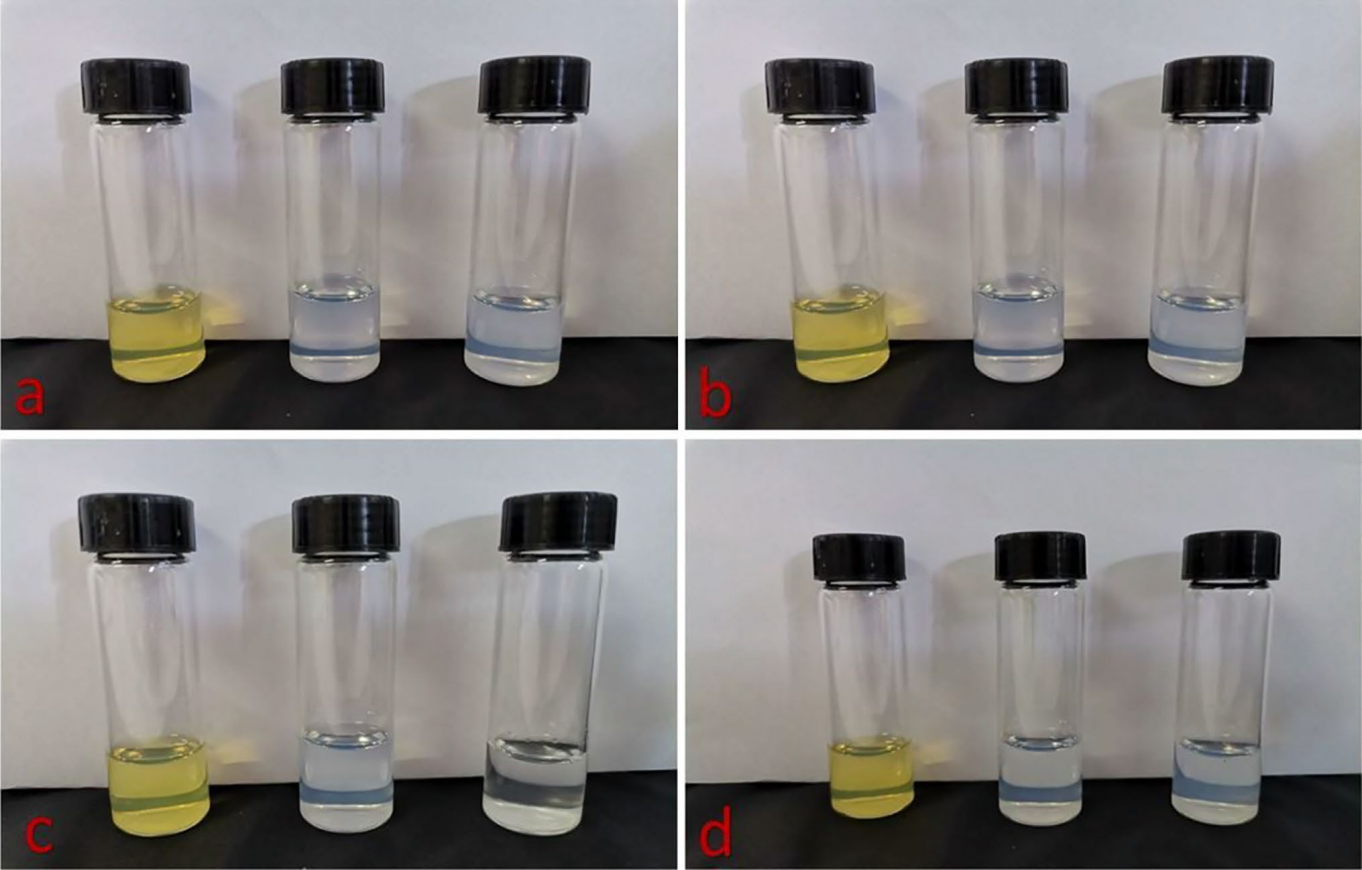
The aspect of Soluplus^®^ micelle aqueous suspensions (from left to right: SNM-IDE, SNM-IBU, and SNM-MIC, respectively) immediately after the preparation (**a**) or after 6 months of storage at 4 °C (**b**), 25 °C (**c**), or 37 °C (**d**)

Furthermore, the macroscopic aspect of the formulations was assessed along the stability assay. From these results, the formulations appeared to remain physically stable (clear and liquid) for up to 6 months at all the three temperature conditions (Fig. 6). Only the SNM-MIC system showed to form a light sediment after 3 months at room temperature and at 4 °C, insinuating that a separation of the drug from the micelles could occur during storage. Conversely, at 37 °C the suspension remained homogenous, most probably because of the positive effect of the temperature on MIC micellar solubility [27] and for the concomitant increase of solubility of MIC with temperature [27, 28].

### Lyophilization study

One important method to obtain stable systems in pharmaceutical technology is freeze-drying. Loaded Soluplus^®^ nanomicelles were thus submitted to lyophilization, with the addition of a cryoprotectant (trehalose) tested at two different concentrations (5 and 15%, w/v). The freeze-drying process enabled to obtain dry powders that, upon reconstitution with water, showed to maintain the micellar mean size than the freshly prepared formulations (Table 7).

**Table 7:** Size analysis (Z-ave) and polydispersity index (PdI) of drug-loaded nanomicelles after lyophilization and redispersion in water

**Formulation**		**SNM-IDE1**	**SNM-IDE2**
**Threalose 5%, w/v**	**Z-AVE (nm) ± S.D**	44.69 ± 0.513	76.63** ± **0.7365
	**Peak1 (nm) ± S.D**	47.96 ± 1.076	84.67 ± 5.138
	**Peak1 area %**	100%	96%
	**PdI ± S.D**	0.061 ± 0.018	0.198 ± 0.015
**Threalose 15%, w/v**	**Z-AVE (nm) ± S.D**	40.38 ± 0.6616	66.53 ± 0.2902
	**Peak1 (nm) ± S.D**	45.20 ± 0.4087	72.42 ± 1.489
	**Peak1 area %**	100%	98%
	**PdI ± S.D**	0.100 ± 0.0023	0.178 ± 0.017

		**SNM-IBU1**	**SNM-IBU2**
**Threalose 5%, w/v**	**Z-AVE (nm) ± S.D**	55.29 ± 0.3889	52.74 ± 0.03669
	**Peak1 (nm) ± S.D**	58.06 ± 0.5200	55.67 ± 1.165
	**Peak1 area %**	100%	100%
	**PdI ± S.D**	0.021 ± 0.021	0.029 ± 0.026
**Threalose 15%, w/v**	**Z-AVE (nm) ± S.D**	54.33 ± 1.027	51.40 ± 0.4450
	**Peak1 (nm) ± S.D**	56.82 ± 0.5757	55.26 ± 1.090
	**Peak1 area %**	100%	100%
	**PdI ± S.D**	0.019 ± 0.012	0.052 ± 0.024

		**SNM-MIC1**	**SNM-MIC2**
**Threalose 5%, w/v**	**Z-AVE (nm) ± S.D**	58.06 ± 0.5412	48.82 ± 0.08418
	**Peak1 (nm) ± S.D**	64.54 ± 3.688	52.85 ± 0.07102
	**Peak1 area %**	100%	100%
	**PdI ± S.D**	0.092 ± 0.07	0.064 ± 0.021
**Threalose 15%, w/v**	**Z-AVE (nm) ± S.D**	60.61 ± 0.035	49.39 ± 0.3092
	**Peak1 (nm) ± S.D**	63.65 ± 0.05194	53.84 ± 0.7087
	**Peak1 area %**	100%	100%
	**PdI ± S.D**	0.041 ± 0.062	0.068 ± 0.019

### In vitrodrug release and release kinetics studies

The in vitro release tests were performed under sink conditions using a 70:30% (v/v) water–methanol mixture as the receiving medium (or water–ethanol for SNM-IDE, thanks to the higher solubility of this drug in ethanol) and followed for 24 h.

**Fig. 7:**
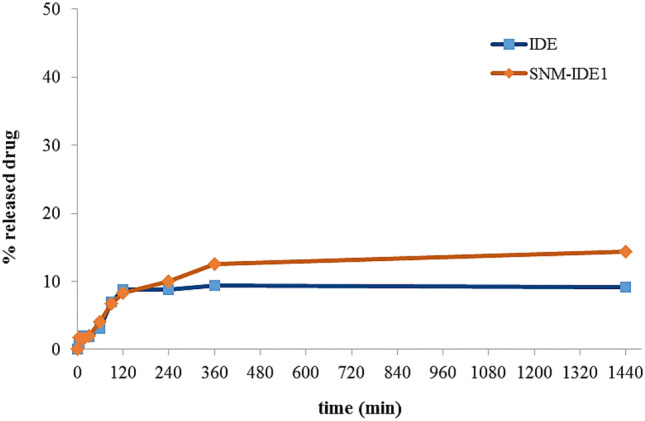
In vitro dissolution profile of IDE from a drug aqueous suspension and SNM-IDE1 micelles

**Fig. 8:**
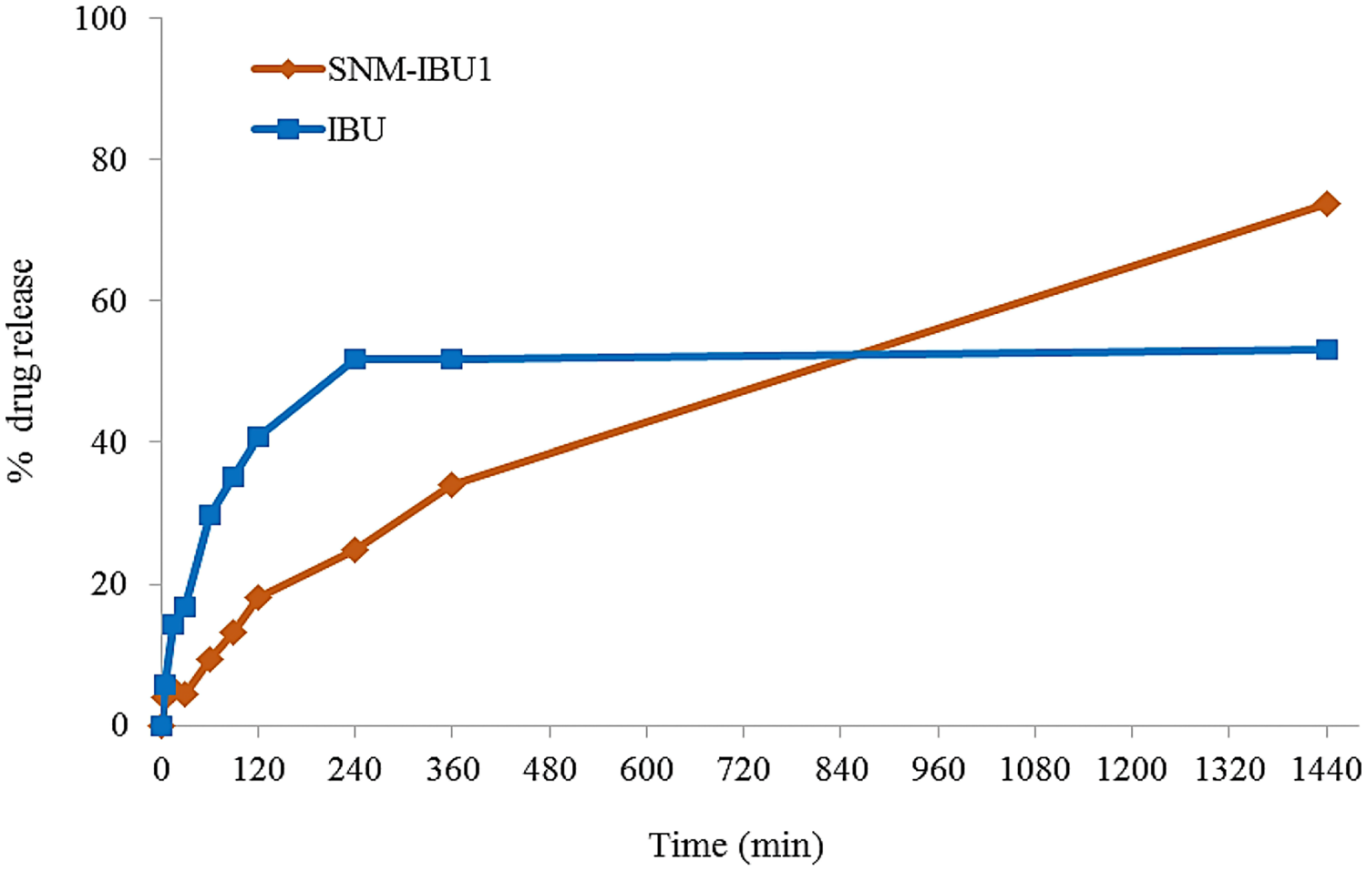
In vitro dissolution profile of IBU from a drug aqueous suspension and SNM-IBU1 micelles

**Fig. 9:**
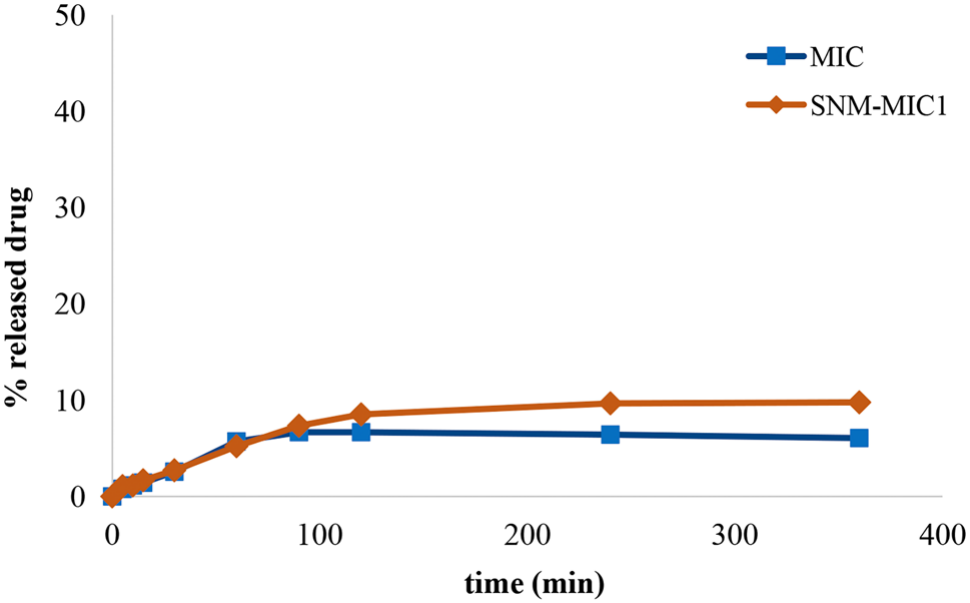
In vitro dissolution profile of MIC from a drug aqueous suspension and SNM-MIC1 micelles

The cumulative release behavior of suspensions of the neat drugs and their nanomicellar formulations are shown in Figs. 7, 8 and 9. The SNM-IDE formulation showed a prolonged release pattern, with about 13% drug release at 24 h. Compared to neat IDE, a maximum value (9%) of dissolved drug was reached from its aqueous suspension after 2 h, with no further increase up to 24 h.

Regarding IBU (Fig. 8), while the neat drug suspension showed a plateau in the dissolution profile after 4 h (at 53% of released drug), the SNM-IBU formulation ensured a linear progressive release of the drug, reaching a 74% value after 24 h.

The cumulative release behavior of suspensions of pure MIC and SNM-MIC is shown in Fig. 9. The SNM-MIC formulation showed about 10% release of the drug at 24 h, at which time only 5% of drug was dissolved from the neat drug suspension. The observed patterns could suggest that the poor solubility of MIC was the limiting parameter in its release from the polymeric nanocarrier.

In summary, the micelle systems loaded with the three tested APIs presented a sustained-release profile, particularly evident in the case of IBU, due to the steady incorporation of these lipophilic molecules within the micelle core. Such a sustained release pattern may ensure a constant concentration of the drugs over time and helps to protract their pharmacological activity.

The above release curves were fitted into different mathematical models (zero-order, first-order, Higuchi, Hixson–Crowell, Weibull, and Korsmeyer–Peppas models) to assess the mechanism of drug release (Table 8). The respective regression coefficient value (*R*^2^) was considered to determine the best fitting model [29].

**Table 8:** Release kinetics parameters obtained from model fitting of in vitro release data of SNM-IBU1, SNM-IDE1, and SNM-MIC1

**Model**	**SNM-IBU1**	**SNM-IDE1**	**SNM-MIC1**
Zero order	0.9696	0.9709	0.9897
First order	0.9660	0.9703	0.9909
Higuchi	0.8914	0.8951	0.9868
Hixson–Crowell	0.9683	0.9624	0.9869
Weibull	0.0684 *b* = 0.2302	0.7703 *b* = 0.7185	0.9505 *b* = 0.9142
Korsmeyer–Peppas	0.8220 *n* = 0.4760	0.7911 *n* = 0.5336	0.9766 *n* = 0.6916

The in vitro release of IBU and IDE from the nanomicelles appeared to follow a zero-order profile, even if other models, i.e., first-order and Hixson–Crowell, gave close *R*^2^ values, suggesting a relevant role of drug dissolution velocity in the observed release profile. Conversely, for these drugs, a low fitting was registered with Higuchi and Korsmeyer–Peppas models, indicating that the drug diffusion through the polymeric matrix was less pertinent; for the latter model, the calculated values of *n* (0.43 < *n* < 0.85) would in particular indicate a non-Fickian transport for spherical systems [30]. Low fitting was also observed with Weibull model, with values of the exponent of time *b* however below or close to 0.75, suggesting a Fickian drug diffusion process [29]. In the case of MIC micelles, a more complex mechanism can be hypothesized, since many models reported a high *R*^2^ value, with a predominance of first-order release. Thus, both diffusion and dissolution mechanisms are involved in MIC release; as a confirmation, the value of *b* close to 1 in Weibull model indicates the presence of a combined mechanism. i.e., Fickian diffusion and case II transport [29].

### Micelle stability upon filtration

All ophthalmic preparations must comply with the sterility requirement, according to the different pharmacopoeias. For liquid formulations, it is possible to apply a filtration under aseptic conditions thanks to appropriate filters having 0.2-µm pore membranes.

In our study, two different filter types, namely PES or hydrophilic PTFE membranes, were selected to determine the more compatible material with the nanomicelles. Experimental results confirmed that, due to the size of nanomicelles, well below the pore cutoff of the used filters, no change in Z-ave and PDI values was registered with both the used sterilizing means (Table 9).

**Table 9:** Size analysis (Z-ave) and polydispersity index (PdI; values ± S.D.) of drug-loaded nanomicelles before and after filtration through a 0.2-μm polyethersulfone (PES) or hydrophilic poly(tetrafluoroethylene) (PTFE) membrane

**Formulation**	**Size analysis**			**PdI ± S.D**
	**Z-Ave (nm)**	**Peak1 (nm)**	**Peak1 area %**	
**SNM-IDE1**				
Unfiltered	59.00 ± 1.0280	63.06 ± 1.145	100	0.053 ± 0.011
PES membrane	58.39 ± 0.9070	58.70 ± 1.161	100	0.039 ± 0.027
PTFE membrane	59.36 ± 0.6447	62.60 ± 0.460	100	0.030 ± 0.012
**SNM-IBU1**	50.20 ± 0.1450	54.22 ± 1.095	99.5	0.164 ± 0.022
Unfiltered	53.70 ± 0.1400	57.03 ± 0.330	100	0.038 ± 0.014
PES membrane	47.03 ± 0.6266	51.28 ± 0.976	100	0.101 ± 0.021
PTFE membrane	50.12 ± 0.7820	53.67 ± 1.367	100	0.030 ± 0.035
**SNM-MIC1**	55.62 ± 0.0396	63.45 ± 3.265	98.8	0.189 ± 0.008
Unfiltered	53.70 ± 0.1400	57.03 ± 0.330	100	0.038 ± 0.014
PES membrane	48.91 ± 0.1266	52.47 ± 0.382	100	0.057 ± 0.014
PTFE membrane	52.69 ± 1.4570	55.95 ± 1.059	100	0.058 ± 0.010

### Micelle stability upon dilution

The capacity of Soluplus^®^ nanomicelles to hold the entrapped drug after dilution in water was evaluated, as a mean to assess their stability after systemic or topical (e.g., ocular) administration, where the contact with physiological fluids, such as tears, can induce a de-aggregation of micellar assembly and a rapid leakage of the entrapped active compound. Two different procedures were followed to confirm the physical stability of the micelles, measuring either the drug UV absorbance or the micelle size after dilution.

**Fig. 10:**
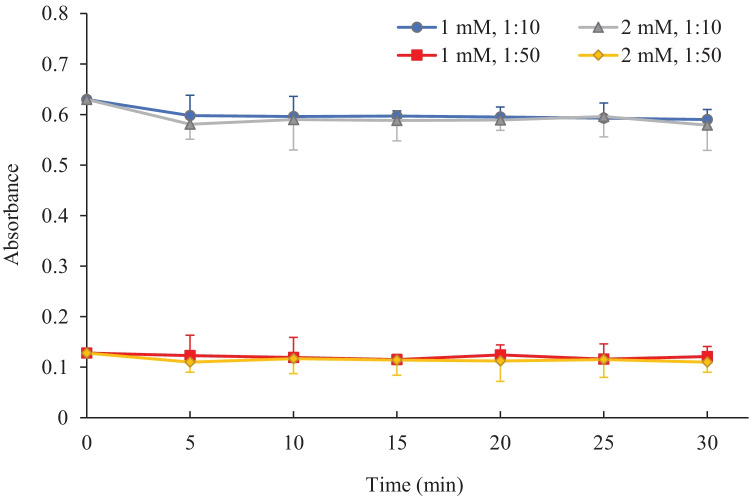
Absorbance of IBU-loaded nanomicelles formulation prepared with 1 or 2 mM Soluplus^®^ after tenfold and 50-fold dilution in water. Each value represents the mean ± S.E. of three separate experiments

Thus, IBU-loaded formulations produced at either 1 or 2 mM Soluplus^®^ concentration were tenfold and 50-fold diluted in water and kept at 35 °C; the UV absorbance of IBU was recorded immediately after and then periodically for 30 min. After a small decrease in the first minutes of incubation, absorbance remained constant along the duration of the experiment, suggesting a good physical stability of the nanomicelles (Fig. 10). Analogous results have been reported for Soluplus^®^ micelles loaded with acyclovir in water and in simulated tear fluid [31] and could be explained by considering the extremely low CMC value of Soluplus (approx. 6.5 × 10^−5^ mM) that resist upon dilution in the nanostructured form.

Analogously, IDE-loaded micelles were diluted and incubated under similar conditions and submitted to PCS analysis to check any change in micelle mean size. As Table 10 shows, such a parameter was not affected, confirming the integrity of the micellar suspensions and their resistance to dilution. The micelles were stable for both Soluplus^®^ concentrations (1 and 2 mM), maintaining a mean size very close to the initial one (T0) for 30 min. The very low PDI registered in all the analyses after the dilution with water was a further demonstration of nanomicelle stability.

**Table 10:** Mean micelle size of IDE-loaded Soluplus^®^ nanomicelles upon tenfold and 50-fold dilution with water and incubation at 35 °C

**Sample (SNM-IDE)**		**Diluted tenfold**		**Diluted 50-fold**	
	**Time (min)**	**Z-Ave (nm) ± SD**	**PDI ± SD**	**Z-Ave (nm) ± SD**	**PDI ± SD**
**1 mM Soluplus**	0	51.22 ± 0.04	0.045 ± 0.013	53.33 ± 2.15	0.058 ± 0.005
	5	51.11 ± 0.09	0.049 ± 0.012	52.99 ± 1.66	0.022 ± 0.009
	10	51.33 ± 0.25	0.049 ± 0.007	52.56 ± 0.90	0.035 ± 0.002
	15	52.36 ± 0.46	0.018 ± 0.009	53.59 ± 0.97	0.069 ± 0.019
	20	52.12 ± 0.58	0.029 ± 0.008	52.82 ± 0.38	0.046 ± 0.017
	25	52.00 ± 0.58	0.049 ± 0.013	53.00 ± 0.84	0.022 ± 0.012
	30	52.14 ± 0.42	0.045 ± 0.037	52.86 ± 1.81	0.068 ± 0.007

**Sample (SNM-IDE)**		**Diluted tenfold**		**Diluted 50-fold**	
	**Time (min)**	**Z-Ave (nm) ± SD**	**PDI ± SD**	**Z-Ave (nm) ± SD**	**PDI ± SD**
**2 mM Soluplus**	0	54.91 ± 0.80	0.038 ± 0.011	54.77 ± 1.39	0.063 ± 0.018
	5	55.75 ± 0.51	0.026 ± 0.012	53.30 ± 0.55	0.036 ± 0.007
	10	55.30 ± 0.75	0.016 ± 0.007	53.08 ± 0.66	0.062 ± 0.034
	15	55.41 ± 0.14	0.037 ± 0.017	53.77 ± 1.48	0.026 ± 0.021
	20	55.22 ± 1.16	0.070 ± 0.035	53.08 ± 0.66	0.047 ± 0.041
	25	56.61 ± 0.51	0.079 ± 0.026	53.74 ± 1.52	0.039 ± 0.030
	30	57.19 ± 0.54	0.043 ± 0.014	53.93 ± 1.04	0.036 ± 0.026

### Viscosity studies

The thickening ability of Soluplus^®^ can be useful to increase the ocular surface permanence time, being an advantage over traditional eye drops that have a contact time with ocular tissue limited to a few minutes [24, 25, 32, 33]. The rheological behavior of Soluplus^®^ aqueous dispersions was thus investigated at 25 °C (room temperature) and 35 °C (ocular surface temperature).

**Fig. 11:**
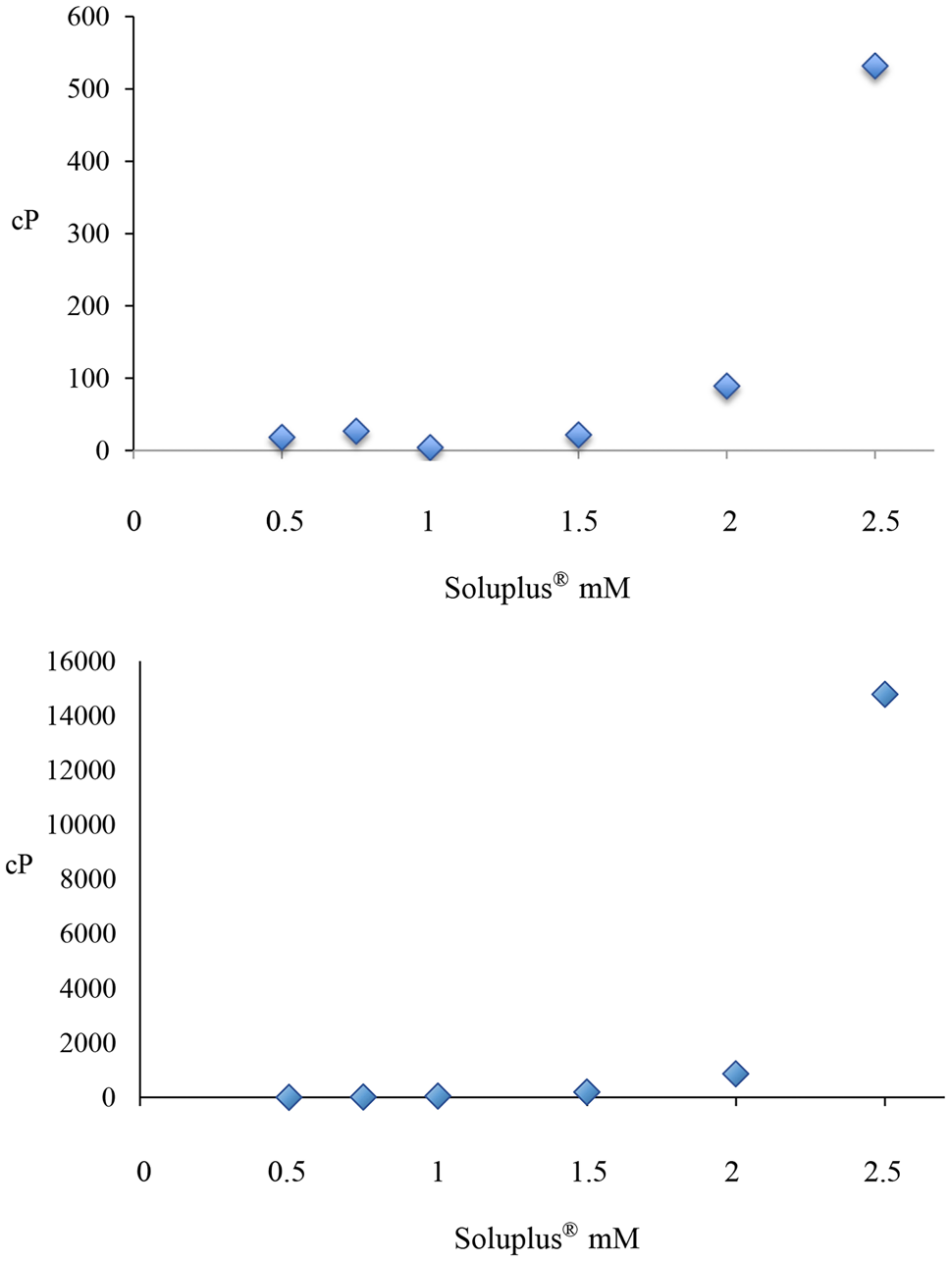
Viscosity values of Soluplus^®^ solutions in water at 25 °C (up) and at 35 °C (down)

Rheological analysis showed that the viscosity of Soluplus^®^ suspensions increased with increasing polymer concentration and at a higher temperature (Table 11 and Fig. 11).

**Table 11:** Viscosity values of aqueous Soluplus^®^ suspensions at different temperatures

**Soluplus**^®^ **concentration (mM)**	**Viscosity**			
	**25 °C**		**35 °C**	
	**(Pa s)**	**(cP)**	**(Pa s)**	**(cP)**
0.5	1.846 · 10^−2^	18.5	1.590 · 10^−3^	1.59
0.75	2.686 · 10^−2^	26.9	6.845 · 10^−3^	6.85
1.0	2.200 · 10^−2^	22.0	3.909 · 10^−2^	39.09
1.5	4.499 · 10^−2^	45.0	1.904 · 10^−1^	190.4
2.0	8.957 · 10^−2^	89.6	8.640 · 10^−1^	864.0
2.5	5.321 · 10^−1^	532.1	14.784	14,784

However, for ophthalmic application, a copolymer concentration above 1.5 mM seems to be unsuitable, since the viscosity of these formulations is clearly above the blur threshold to be applied as eye-drops [32]. Higher Soluplus^®^ concentrations could however remain interesting to be investigated for the production of mucoadhesive gelling systems. In fact, at high concentrations, the micellar suspension can be converted into a weak gel on the ocular surface, which may prolong the permanence time of the formulation, offering soft resistance against blinking and eventually a lubricating effect [33–35]. The observed behavior is in accordance with literature [25], which reported that a 2 mM Soluplus^®^ solution having a gelling point of 39 °C. Soluplus^®^ formulations may thus offer the advantage of using a single polymer material for affording both the nanomicelles and gelling properties.

## Conclusions

Soluplus^®^ is a graft amphiphilic copolymer that is frequently used as an excipient in solid dosage forms as a dissolution and a solubility enhancer [36–39].

The aim of this study was to prepare and characterize Soluplus^®^ nanomicelles for enhancing the solubility of three APIs (idebenone, ibuprofen, and miconazole) belonging to class II of the Biopharmaceutics Classification System (BCS), meaning that they exhibit good permeability but poor solubility.

Drug-loaded Soluplus^®^ micelles were prepared by two different methods: direct dissolution and solvent evaporation-thin film rehydration. Their characterization showed dimensions appropriate for an ocular instillation, with a mean size lower than 200 nm and a very high size homogeneity. All the drug-loaded micelles were stable at room temperature, at 4 and 37 °C up to 3 months. A preliminary lyophilization test was carried out, in the presence of a cryoprotectant, to assess this further possibility of enhancing over time the storability of nanomicelles, an appealing aspect for industrial development. Soluplus^®^ nanomicelles can be also easily sterilized by membrane filtration (0.2-µm PES or PTFE membranes) without significant size changes.

Solubility studies showed that the solubility of the tested APIs increased linearly with the concentration of Soluplus^®^; in the case of IDE, a plateau in drug solubility was reached at a copolymer concentration of 2 mM. Furthermore, from the viscosity studies, these Soluplus^®^ micelles confirmed the potential to be exploited in applications where the use of a bioadhesive material is desirable, such as topical ocular administration.

In conclusion, Soluplus^®^ drug-loaded nanomicelles are a valid means to improve the solubility of BCS-class II drugs and, because of their physical features and stability, can be investigated as a potential carrier for topical and systemic drug delivery.

## Supplementary Information

Below is the link to the electronic supplementary material.

Supplementary file1 (DOCX 30 KB)
