# Supplementary material for: Soluplus® polymeric nanomicelles improve solubility of BCS-class II drugs
Source: Drug Deliv Transl Res. 2022 May 23;12(8):1991–2006. doi: 10.1007/s13346-022-01182-x (PMC9242938; doi:10.1007/s13346-022-01182-x)
Supplement: Supplementary file 1 — Supplementary file1 (DOCX 30 KB) [file 13346_2022_1182_MOESM1_ESM.docx]

**PIGNATELLO ET AL.**

**SUPPLEMENTARY MATERIAL:**

Table S1. Stability of IDE-loaded nanomicelles under various storage conditions.

|  |  |  | **SNM-IDE1** | **SNM-IDE2** |
| --- | --- | --- | --- | --- |
|  |  |  |  |  |
| **1 MONTH** | **4°C** | **Z-AVE** | 48.84±0.558 | 57.90±0.291 |
|  |  | **PdI** | 0.091±0.01 | 0.052±0.04 |
|  | **R.T.** | **Z-AVE** | 42.00±0.617 | 57.38±0.692 |
|  |  | **PdI** | 0.087±0.01 | 0.063±0.03 |
|  | **37°C** | **Z-AVE** | 59.97±0.901 | 61.23±0.280 |
|  |  | **PdI** | 0.015±0.01 | 0.077±0.04 |
| **3 MONTHS** | **4°C**  **R.T.**  **37°C** | **Z-AVE** | 57.69±0.761 | 58.33±0.576 |
|  |  | **PdI** | 0.011±0.07 | 0.028±0.02 |
|  |  | **Z-AVE** | 59.01±0.717 | 57.38±0.692 |
|  |  | **PdI** | 0.028±0.01 | 0.063±0.03 |
|  |  | **Z-AVE** | 60.31±1.021 | 57.79±0.761 |
|  |  | **PdI** | 0.033±0.01 | 0.011±0.01 |
| **6 MONTHS** | **4°C** | **Z-AVE** | 64.59±2.022 | 61.23±0.280 |
|  |  | **PdI** | 0.092±0.05 | 0.077±0.04 |
|  | **R.T.** | **Z-AVE** | 58.85±0.323 | 57.90±0.291 |
|  |  | **PdI** | 0.027±0.02 | 0.052±0.05 |
|  | **37°C** | **Z-AVE** | 61.65±1.145 | 57.58±0.778 |
|  |  | **PdI** | 0.085±0.02 | 0.033±0.04 |

Table S2. Stability of IBU-loaded nanomicelles under various storage conditions.

|  |  |  |  | **SNM-IBU1** | **SNM-IBU2** |
| --- | --- | --- | --- | --- | --- |
|  |  |  |  |  |  |
| **1 MONTH** | **4°C** | **Z-AVE** | | 55.85±0.4697 | 51.16± 0.2079 |
|  |  | **PK1** | | 60.65±1.211 | 55.58±1.850 |
|  |  | **A%** | | 100% | 100% |
|  |  | **PdI** | | 0.070±0.0029 | 0.064±0.035 |
|  | **R.T.** | **Z-AVE** | | 52.02±0.8271 | 53.67±7.817 |
|  |  | **PK1** | | 55.75±2.423 | 44.33±14.87 |
|  |  | **A%** | | 100% | 100% |
|  |  | **PdI** | | 0.054±0.06 | 0.393±0.526 |
|  | **37°C** | **Z-AVE** | | 51.00±1.104 | 48.68±0.4804 |
|  |  | **PK1** | | 54.97±2.201 | 52.81±1.027 |
|  |  | **A%** | | 100% | 100% |
|  |  | **PdI** | | 0.054±0.029 | 0.070±0.009 |
|  |  |  | |  |  |
| **3 MONTHS** | **4°C** | **Z-AVE** | | 48.66±0.1629 | 54.38±1.487 |
|  |  | **PK1** | | 52.40±1.209 | 57.92±2.089 |
|  |  | **A%** | | 100% | 100% |
|  |  | **PdI** | | 0.106±0.037 | 0.046±0.020 |
|  | **R.T.** | **Z-AVE** | | 46.81±0.5928 | 55.28±0.5002 |
|  |  | **PK1** | | 49.68±1.210 | 57.87±1.912 |
|  |  | **A%** | | 100% | 100% |
|  |  | **PdI** | | 0.038±0.045 | 0.082±0.068 |
|  | **37°C** | **Z-AVE** | | 47.09±1.152 | 57.52±2.078 |
|  |  | **PK1** | | 50.89±2.832 | 59.87±1.152 |
|  |  | **A%** | | 100% | 100% |
|  |  | **PdI** | | 0.11±0.066 | 0.058±0.054 |

| **6 MONTHS** | **4°C** | **Z-AVE** | 49.65±0.1444 | 53.11±0.997 |
| --- | --- | --- | --- | --- |
|  |  | **PK1** | 49.20±1.119 | 53.92±1.489 |
|  |  | **A%** | 100% | 99% |
|  |  | **PdI** | 0.116±0.088 | 0.106±0.033 |
|  | **R.T.** | **Z-AVE** | 48.11±0.5444 | 57.00±0.4743 |
|  |  | **PK1** | 49.18±1.445 | 57.66±1.334 |
|  |  | **A%** | 100% | 100% |
|  |  | **PdI** | 0.067±0.011 | 0.098±0.012 |
|  | **37°C** | **Z-AVE** | 51.09±1.221 | 59.11±1.777 |
|  |  | **PK1** | 50.87±1.445 | 61.33±1.009 |
|  |  | **A%** | 100% | 99% |
|  |  | **PdI** | 0.103±0.044 | 0.097±0.098 |

Table S3. Stability of MIC-loaded nanomicelles under various storage conditions.

|  |  |  |  | **SNM-MIC1** | **SNM-MIC2** |
| --- | --- | --- | --- | --- | --- |
|  |  |  |  |  |  |
| **1 MONTH** |  | **4°C** | **Z-AVE** | 53.27±0.9853 | 43.78±0.5460 |
|  |  |  | **PK1** | 56.19±0.9306 | 48.04±0.7358 |
|  |  |  | **A%** | 100% | 100% |
|  |  |  | **PdI** | 0.030±0.031 | 0.082±0.015 |
|  |  | **R.T.** | **Z-AVE** | 49.64±0.5220 | 48.95±1.026 |
|  |  |  | **PK1** | 53.45±1.252 | 53.62±1.489 |
|  |  |  | **A%** | 100% | 100% |
|  |  |  | **PdI** | 0.1±0.028 | 0.088±0.016 |
|  |  | **37°C** | **Z-AVE** | 56.29±0.5479 | 46.73±0.7346 |
|  |  |  | **PK1** | 60.58±1.345 | 50.09±0.18 |
|  |  |  | **A%** | 100% | 100% |
|  |  |  | **PdI** | 0.075±0.061 | 0.050±0.022 |

|  |  |  | **SNM-MIC1** | **SNM-MIC2** |
| --- | --- | --- | --- | --- |
|  |  |  |  |  |
| **3 MONTHS** | **4°C** | **Z-AVE** | 50.90±1.052 | 43.81±1.029 |
|  |  | **PK1** | 54.73±2.208 | 46.95±2.285 |
|  |  | **A%** | 100% | 100% |
|  |  | **PdI** | 0.084±0.068 | 0.053±0.046 |
|  | **R.T.** | **Z-AVE** | 59.86±1.087 | 43.04±0.2122 |
|  |  | **PK1** | 62.51±1.609 | 43.54±0.9064 |
|  |  | **A%** | 99.5% | 100% |
|  |  | **PdI** | 0.058±0.074 | 0.113±0.011 |
|  | **37°C** | **Z-AVE** | 64.89±1.781 | 53.55±0.4384 |
|  |  | **PK1** | 71.55±2.190 | 56.87±0.5415 |
|  |  | **A%** | 100% | 100% |
|  |  | **PdI** | 0.080±0.026 | 0.071±0.028 |

|  |  |  | **SNM-MIC1** | **SNM-MIC2** |
| --- | --- | --- | --- | --- |
|  |  |  |  |  |
| **6 MONTHS** | **4°C** | **Z-AVE** | 52.11±1.300 | 49.11±0.929 |
|  |  | **PK1** | 54.11±1.887 | 52.00±2.005 |
|  |  | **A%** | 100% | 99.3% |
|  |  | **PdI** | 0.056±0.012 | 0.039±0.077 |
|  | **R.T.** | **Z-AVE** | 57.69±1.111 | 53.04±0.8770 |
|  |  | **PK1** | 60.53±1.445 | 49.44±0.8871 |
|  |  | **A%** | 99.5% | 100% |
|  |  | **PdI** | 0.097±0.023 | 0.099±0.033 |
|  | **37°C** | **Z-AVE** | 62.89±1.443 | 56.56±0.4365 |
|  |  | **PK1** | 73.00±2.007 | 56.99±0.443 |
|  |  | **A%** | 99.5% | 99.7% |
|  |  | **PdI** | 0.102±0.044 | 0.097±0.022 |
